# Machine learning in the estimation of CRISPR-Cas9 cleavage sites for plant system

**DOI:** 10.3389/fgene.2022.1085332

**Published:** 2023-01-09

**Authors:** Jutan Das, Sanjeev Kumar, Dwijesh Chandra Mishra, Krishna Kumar Chaturvedi, Ranjit Kumar Paul, Amit Kairi

**Affiliations:** ^1^ ICAR-Indian Agricultural Research Institute, New Delhi, India; ^2^ ICAR-Indian Agricultural Statistics Research Institute, New Delhi, India

**Keywords:** CRISPR, Cas9, SgRNA, genome editing, off-target, artificial neural network, support vector machine, random forest

## Abstract

CRISPR-Cas9 system is one of the recent most used genome editing techniques. Despite having a high capacity to alter the precise target genes and genomic regions that the planned guide RNA (or sgRNA) complements, the off-target effect still exists. But there are already machine learning algorithms for people, animals, and a few plant species. In this paper, an effort has been made to create models based on three machine learning-based techniques [namely, artificial neural networks (ANN), support vector machines (SVM), and random forests (RF)] for the prediction of the CRISPR-Cas9 cleavage sites that will be cleaved by a particular sgRNA. The plant dataset was the sole source of inspiration for all of these machine learning-based algorithms. 70% of the on-target and off-target dataset of various plant species that was gathered was used to train the models. The remaining 30% of the data set was used to evaluate the model’s performance using a variety of evaluation metrics, including specificity, sensitivity, accuracy, precision, F1 score, F2 score, and AUC. Based on the aforementioned machine learning techniques, eleven models in all were developed. Comparative analysis of these produced models suggests that the model based on the random forest technique performs better. The accuracy of the Random Forest model is 96.27%, while the AUC value was found to be 99.21%. The SVM-Linear, SVM-Polynomial, SVM-Gaussian, and SVM-Sigmoid models were trained, making a total of six ANN-based models (ANN1-Logistic, ANN1-Tanh, ANN1-ReLU, ANN2-Logistic, ANN2-Tanh, and ANN-ReLU) and Support Vector Machine models (SVM-Linear, SVM-Polynomial, SVM-Gaussian However, the overall performance of Random Forest is better among all other ML techniques. ANN1-ReLU and SVM-Linear model performance were shown to be better among Artificial Neural Network and Support Vector Machine-based models, respectively.

## 1 Introduction

Genome editing (or gene editing) is nothing but the deletion, insertion and replacement of DNA at an explicit site in the genome of any organism. Molecular scissors, also known as designed nucleases, is used in the molecular laboratory to alter gene functions by editing or by modification of part of DNA (Urnov et al., 2010; Perez-Pinera et al., 2012).

Although there are many different gene editing methods (such as CRISPR-Cas9, ZFNs or TALENs etc.) available. Though techniques have been extensively used in a wide variety of cells, tissues and organisms (Sander and Joung 2014; Ma et al., 2017; Musunuru 2017) but CRISPR-Cas9 is the most widely used method by researchers worldwide.

CRISPR, Clustered Regularly Interspaced Short Palindromic Repeats, is condensed segments of bacterial DNA that contain repetitive base sequences. It plays a critical role in providing natural immunity to bacteria against foreign DNA. With the event of identification of any viral DNA, the bacterium produces guide RNA, two strands of short RNA. Then, it forms a complex with an endonuclease enzyme, which is named Cas9 (CRISPR Associated Protein 9) (Barrangou et al., 2007; Terns and Terns 2011). The CRISPR-Cas9 complex targets and cuts out the viral DNA rendering the virus disabled. The Cas9 nuclease will not bind to the DNA if the target sequence is not followed by the Protospacer Adjacent Motif, or PAM, which helps the enzyme distinguish between the bacterial DNA and the viral DNA target. The CRISPR-Cas9 system then has the ability to store this viral data so that it will be able to recognize and eliminate future viral threats. CRISPRs are generally found in roughly 50% and 90% of sequenced genomes of bacteria and archaea, respectively (Sander and Joung 2014; Westra et al., 2014; Bortesi and Fischer 2015; Ma et al., 2017; Musunuru 2017).

The flexibility of the CRISPR-Cas9 system and its ability to find and modify particular genes can be used in research in the field of medicine, drug discovery and agriculture. The recent discovery of sequence-based genome editing technology for crop improvement (Georges and Ray, 2017). Particularly, CRISPR-Cas9 has shown the potential to address the emerging challenges of crop science and agriculture. This technology is capable of modifying any genomic sequence and can result in desired traits in organisms including crop species provided that the protospacer adjacent motif (PAM) sequence is available. CRISPR-Cas9 is an efficient, cost-effective, easier and highly precise genome editing tool as compared to other genome editing tools *viz.* zinc finger nucleases (ZFNs) and transcriptional activator-like effector nucleases (TALENs) (Wood et al., 2011). With the introduction and demonstration of the CRISPR-Cas9 system in 2012, it has been widely accepted among researchers across the globe. This genome editing system has been widely used and targeted many important genes of various cell lines and organisms, including bacteria, *C. elegans*, *Xenopus tropicalis*, yeast, zebrafish, *Drosophila*, rabbits, plants, monkeys, humans, rats and mice. Several workers have used this method and introduced single-point mutations, either deletions or insertions, into a target gene by using sgRNA (Sander and Joung 2014). Thus, CRISPR-Cas9 is one of the most emerging technology in the editing of plant genomes to cope up with emerging challenges of agriculture due to climate change and food security (Sovová et al., 2016; Haque et al., 2018).

Though sgRNA aims to target a specific segment of DNA, sometimes it is attached to other sites of DNA and unfortunately causes off-target mutations. Again it can tolerate mismatches in sgRNA-DNA at different positions but at the same time sensitive to the position, number and distribution of mismatches. Alter gene functions led by these off-target mutations can cause major genomic instability and pose a major threat while using CRISPR-Cas9 gene editing (Cho et al., 2014). It is imperative that altered but untargeted gene functions caused by off-targeted gene mutation lead to Genomic instability; it is one of the major problems associated with the application of CRISPR-Cas9 gene editing (Hsu et al., 2013; Cho et al., 2014). One way to safeguard from the ill effects of gene editing is to accurate prediction of off-target sites of the genome. Though, there are many off-target prediction methods available that works on the principles of calculation of scores based on the positions of the mismatches to the guide sequence (Haeussler et al., 2016; Xu et al., 2017). The score of each base pair in sgRNA-DNA is imitatively using the statistical analysis (Pearson correlation coefficient) of the mismatch effects based on prior gene editing experiments. Most of the current off-target prediction methods calculate scores, based on the positions of the mismatches to the guide sequence (Haeussler et al., 2016). The score of each base pair in sgRNA-DNA is imitatively using the statistical analysis of the mismatch effects based on prior gene editing experiments (Haeussler et al., 2016; Xu et al., 2017). For example, CCTop considers the distance of the mismatch from the PAM site when evaluating the specificity of candidate sgRNAs, “Optimized CRISPR Design” incorporates a position-specific mismatch penalty and additionally considers the spatial distribution of mismatches, and the CFD score penalizes each mismatch according to its specific substitution type and position (Zhang et al., 2014), MIT score only considered the positions and counts of the mismatched sites of sgRNA-DNA as the features to score the potential off-targets (Hsu et al., 2013) and the CFD score penalizes each mismatch according to its specific substitution type and position (Doench et al., 2016). Importantly, while these and other widely-used methods have been developed based on empirical data they mostly neglect the genomic context surrounding the target sequence and instead focus on predicting off-target effects for a given sgRNA using basic sequence features. It is significant that, even though these and other widely-used approaches were developed using empirical data, they primarily ignore the genomic context around the target sequence and instead concentrate on forecasting off-target effects for a given sgRNA using simple sequence properties (Sanjana et al., 2014). For accurately predicting cleavage sites, a variety of machine learning and deep learning method-based tools are available for humans (Abadi et al. 2017; Lin and Wong 2018) and plants (Hesami et al. 2021; Niu et al. 2021). These tools incorporate a wide range of features, including those that are specific to the genomic target, features that explain the sgRNA’s thermodynamics, and features about the pair-wise similarity between the sgRNA and the genomic target. To precisely determine the cleavage location of a gene, machine learning techniques are therefore quite advanced and effective.

Machine Learning is considered a subset of computational or Artificial Intelligence and provides the capacity for computers to learn from data without being explicitly programmed (Pedregosa, 2011). Compared to the other programming languages, it doesn’t have explicit and defined steps or conditions to solve the problem. Rather, it enables to fit of the programs, algorithms, or methods to learn a specific task from the experimental data set (Mitchell et al., 2003). These trained models help the machine to take decisions on different and variable situations based on the learning upon a dataset. Machine learning has been widely used in different fields of plant science such as plant breeding (van Dijk et al., 2021), *in vitro* culture (Hesami and Jones 2020), stress phenotyping (Singh et al., 2016), stress physiology (Jafari and Shahsavar 2020), plant system biology (Hesami et al., 2022), plant identification (Grinblat et al., 2016), and pathogen identification (Mishra et al., 2019).

The currently available machine learning- or deep learning-based algorithms for CRISPR off-target prediction are mostly based on data either from animal or human genomes. Their effectiveness on plant genomes has not been widely demonstrated. As a result, we used plant data to create machine learning-based models for plant genomes. The development of machine learning-based models for the prediction of CRISPR-cas9 off-target sites for plant genomes and for assessing the effectiveness of these models were the key contributions made in this study.

## 2 Materials and methods

### 2.1 Data collection

A thorough literature review has been conducted to gather information on off-target and on-target sequences, as well as associated sgRNA sequences, specific to crop species. We used Google Scholar as a search engine to look up published and accessible literature using terms like “off-target sites in crops,” “off-target estimation,” “CRISPR-Cas9 on-target and off-target sites,” “off-target effect minimization in a plant cell,” etc. Search results from the last 5 years were used to choose a few research papers that describe CRISPR-Cas9 experiments conducted on various crop species. Then, wherever it was available, the genomic sequence of the sgRNA, the on-targets, and the off-target sites were collected from the shortlisted articles.

### 2.2 Data preparation

A computer program was created in the Python programming language to extract the parameters from a large number of sequences based on the pairwise alignment of sgRNA and genome target sites, features regarding the nucleotide contents of 20 nucleotide sites and their contiguous genomic regions, and features regarding the PAM sites and nearby the nucleotides (Abadi et al., 2017). Then, using the constructed program, features based on the aforementioned criteria were extracted from the sequences of sgRNA, on-targets, and off-target sites of the genome. The creation of classification models based on machine learning uses these extracted characteristics as explanatory variables. The related site-specific on-target and off-target information were used to create a response variable, where respective on-targets were labeled as 1 and off-targets as 0.

### 2.3 Machine learning models/experiments

Recent machine learning-based classification modeling techniques have been employed to create robust classification models. The Artificial Neural Network (ANN), Support Vector Machine (SVM), and Random Forest machine learning techniques have all been investigated. In the process of creating a viable model, a variety of different variations and structures connected to the above modeling paradigm have also been tested.

#### 2.3.1 Artificial neural network

Two Multi-layered perceptrons (MLP) structures (Chatterjee et al., 2022) of ANN were chosen with three and four hidden layers and named ANN1 and ANN2 respectively. The layer-wise number of perceptron was arbitrarily taken as 25:25:25 for each layer in ANN1 whereas 30:20:10:5 for each of the four layers in ANN2, starting from the input layer to the output layer. To train the above ANN models, three different activation functions have been considered here. They are Logistic, Tanh and ReLu, thus altogether six ANNs, namely ANN1-Logistics, ANN1-Tanh, ANN1-ReLu, ANN2-Logistics, ANN2-Tanh, and ANN2-ReLu, were used to model the training data. The MLPClassifier implemented in the python Scikit-learn module (Pedregosa, 2011) was used for the training of the ANN models using training data set. The following hyper-parameters of MLPClassifier were used during the training. To validate the model 5-fold cross-validation techniques (Refaeilzadeh et al., 2009) were used. The following parameters were used for developing the model.

**hidden_layer_sizes:** 25:25:25 and 30:20:10:5.

**Activation:** Used three activation functions i.e., logistic, tanh and relu.

**Solver:** Adam solver was used for optimizing the weights.

**learning_rate_init:** Used initial learning rate as 0.001.

#### 2.3.2 Support vector machine

Depending on the type of kernel function used, four SVM models are developed, which are named as SVM-Linear, SVM-Polynomial, SVM-Gaussian and SVM-Sigmoid. The Support Vector Classifier (SVC) implemented in the python Scikit-learn module (Pedregosa, 2011) was used for training the SVM models using training data set. Polynomial kernel-based SVM model used with the degree of 3. Here also, 5-fold cross-validation techniques were used for validation of the model.

#### 2.3.3 Random forest

For the training dataset and a given set of features, we implemented the RF model using RandomForestClassifier implemented in the python scikit-learn module (Pedregosa, 2011). We used 5-fold cross-validation techniques for the validation of our model. In this study, we used it for 100 estimators and used the Gini index to measure the quality of a split and for building the trees, a bootstrap method was used.

## 4 Results

### 4.1 Crop species wise sgRNA related on-target and off-target sequence data

The research article containing sequence details about sgRNA related to on-target and off-target data was discovered in the following journals: Frontiers in plant science, Nature, Scientific Reports, Plant Biotechnology Journal, PloS one, Molecular plant pathology, Journal of Genetics and Genomics, Nucleic acids research, Nature communications, Rice, Molecular plant, Plant cell reports, Nature Biotechnology, Nature protocols, Cell, Journal of molecular biology, Journal of Molecular Biology, and Journal of Molecular Medicine. From a Google Scholar search using the stated keyword, a total of 64 research publications were found. Thirty two research publications detail the sequence of a crop-specific sgRNA and its associated on-target and off-target regions (Table 1). Table 1 perusal reveals that 15 important crop species were used to gather the sequencing data for 51 sgRNA and the related 174 and 205 on-targets and off-targets sites, respectively. SgRNA was between 17 and 20 nucleotides in length. The average length of the off-target and on-target was determined to be 23 or longer nucleotides. As a result, 51 sgRNA were the subject of a total of 379 data points collection, together with the matching on-target and off-target locations. The values of 48 explanatory variables and one response variable were obtained using a Python program. The complete 379-point data set, which contained 48 variables, was divided into two halves by chance, with 265 and 114 entries for each of the 31 variables being used for model building and model evaluation, respectively.

**TABLE 1:** Crop wise number of sgRNA, on-target and off-target.

Sl. No.	Crops name	No. of sgRNA	No. of on-target	No. of off-target	References
1	Rice	8	40	36	Li et al. (2016), Li et al. (2017b), Wang et al. (2017), Xu et al. (2014), Xu et al. (2015), Zhou et al. (2014)
2	Wheat	5	19	30	Shan et al. (2014), Zhang et al. (2016), Kim et al. (2018)
3	Soybean	2	10	5	Cai et al. (2015), Sun et al. (2015)
4	Cotton	8	20	33	Chen et al. (2017), Gao et al. (2017), Li et al. (2017a), Wang et al. (2018)
5	Cucumber	2	2	5	Chandrasekaran et al. (2016)
6	Tobacco	2	6	5	Gao et al. (2014)
7	Strawberry	2	5	7	Martín-Pizarro et al. (2019)
8	Watermelon	2	2	2	Tian et al. (2016)
9	Tomato	4	10	13	Brooks et al. (2014), Čermák et al. (2015), Pan et al. (2016)
10	Grape	2	8	10	Nakajima et al. (2017)
11	Potato	2	5	3	Butler et al. (2015), Wang et al. (2015), Andersson et al. (2017)
12	Apple	1	6	4	Malnoy et al. (2016)
13	Orange	4	15	20	Jia and Nian (2014)
14	Maize	6	31	23	Feng et al. (2016), Svitashev et al. (2016), Feng et al. (2018)
15	Barley	1	5	9	Kapusi et al. (2017)
Total		51	174	205	

### 4.2 Importance of features

In addition to improving prediction abilities, the learning strategy enabled researchers to thoroughly comprehend the most crucial Cas9 traits. When the entire set of features was evaluated, three clusters emerged among the top 30 features: 1) a pair-wise similarity characteristic between the sgRNA and the DNA site Along with the pair-wise alignment score, the number of mismatches, the number of RNA/DNA bulges, and the kind of mismatch (transversion, transversion, or wobble) were all included in this cluster. 2) GC content, DNA enthalpy (Breslauer et al., 1986), and several measures of DNA spatial structure, such as minor groove width and bending stiffness (Zhou et al., 2013), were among these. These characteristics are related to the nucleotide composition of the 20-nt location and the genomic region surrounding it. 3) The DNA geometry scores calculated in and around this region, as well as PAM site characteristics like the PAM type (NGG or NAG), were among them. In this investigation, 30 feature numbers (Table 2) were used (Abadi et al., 2017).

**TABLE 2:** List of all the features used in this study.

Features derived from pair-wise sequence alignment	Features derived from nucleotide contents	Features derived from PAM sites
■ Pair-wise alignment score	■ 20th position nucleotide	■ PAM type
■ Wobble total	■ MGW (minor groove width) at the PAM NNGGN	■ In exon (non-NGG strand)
■ RNA bulges	■ DNA enthalpy—extended 223 nt	■ Downstream nt—position 1
■ Mismatches in positions 17–20	■ Nucleotide—position 2	■ Downstream nt—position 5
■ Mismatches	■ DHS (DNAse hypersensitive site) signal value	■ Downstream nt—position 2
■ DNA bulges	■ Guanine occupancy	■ In exon (NGG strand)
■ Tv (transversion mismatches) total	■ Distance from nucleosome	■ NGG strand expression
■ RR (purine-purine) total	■ Nucleotides—positions 4–5	■ Non-NGG strand expression
■ YY (pyrimidine-pyrimidine) total	■ Transcription region	■ PAM N nucleotide
	■ Coding region	
	■ GC content—extended	
	■ Nucleotide—position 4	

### 4.3 Model results

Results from different machine learning-based developed models and their comparisons in various aspects are given below-

#### 4.3.1 Artificial neural network model

**FIGURE 1:**
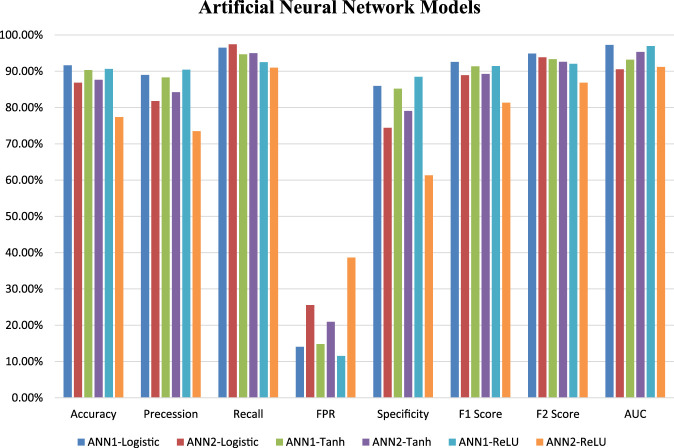
Graphical representation of six ANN model’s performance based on a different statistical measure.

**FIGURE 2:**
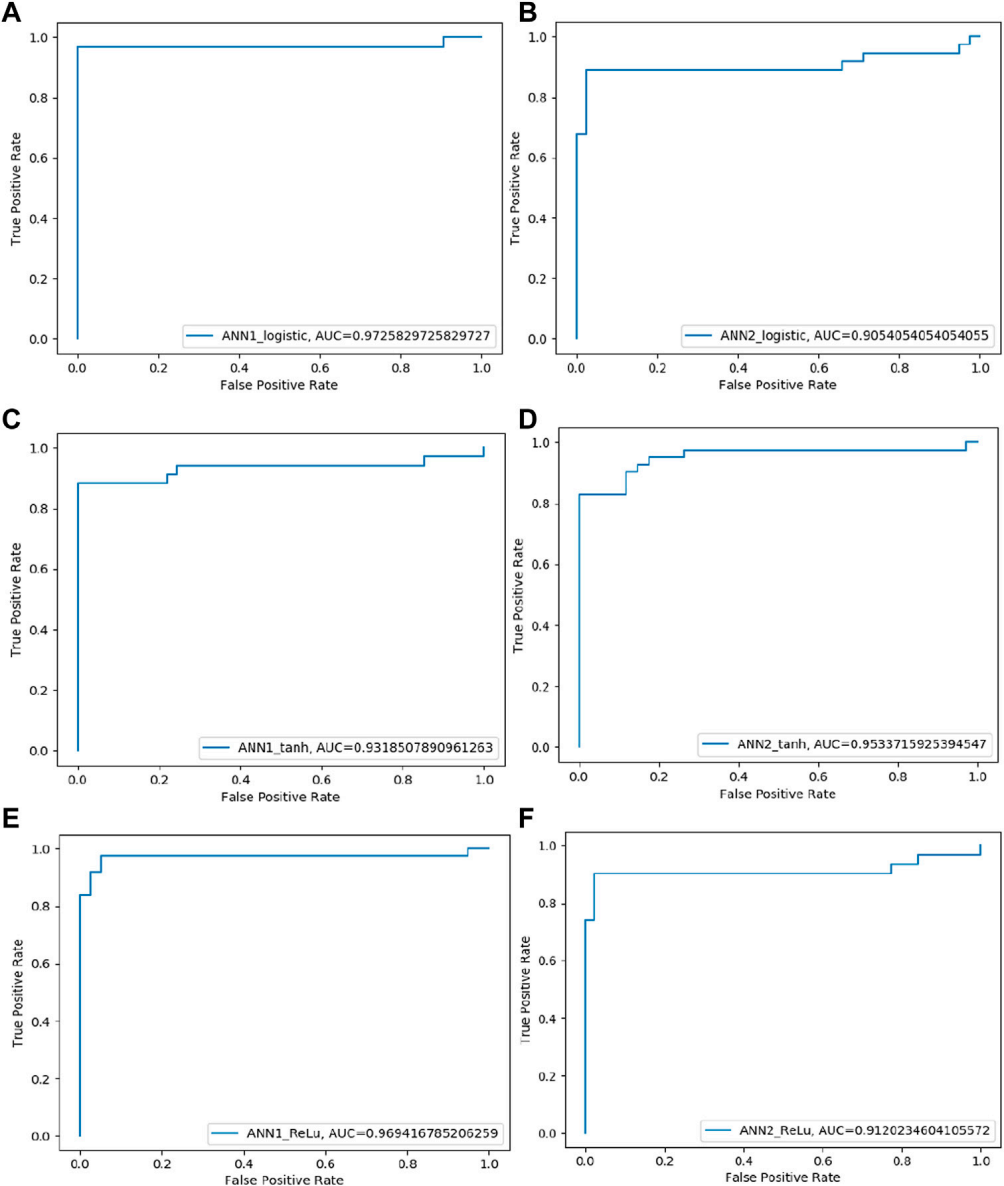
ROC curve of four SVM models performance based on AUC score: **(A)** SVM-Linear model ROC curve **(B)** SVM-Polynomial model ROC curve **(C)** SVM-rbf model ROC curve **(D)** SVM-Sigmoid model ROC curve.

Six ANN models were trained using training datasets, and the effectiveness of each trained model was assessed using a variety of evaluation parameters on the test dataset. In this research, we used k-Fold, or *k* = 5 cross-validation approaches, for validation purposes. All six evaluation parameter values have been shown against the implemented ANN models (Table 3), which makes it simple to grasp similar comparisons in a graphical style (Figures 1, 2). This study is based on the Technique for Order Preference by Similarity to Ideal Solution (TOPSIS) (Hwang and Yoon 1981). From six ANN-based models, ANN1-Logistic obtained a TOPSIS analysis score of 0.85134 and is ranked one (Supplementary Material).

**TABLE 3:** Performance evaluation of developed six ANN models on plant dataset based on accuracy, precession, recall, FPR, specificity, F1 score, F2 score and AUC (figures in percentage).

Models	Accuracy	Precession	Recall	FPR	Specificity	F1 score	F2 score	AUC
ANN1-Logistic	91.65	89.00	96.49	14.05	85.95	92.59	94.89	97.26
ANN2-Logistic	86.87	81.78	97.44	25.57	74.43	88.93	93.85	90.54
ANN1-Tanh	90.33	88.29	94.68	14.80	85.20	91.37	93.33	93.18
ANN2-Tanh	87.66	84.23	94.98	20.95	79.05	89.28	92.61	95.34
ANN1-ReLU	90.65	90.44	92.49	11.52	88.48	91.45	92.07	96.94
ANN2-ReLU	77.39	73.50	91.00	38.65	61.35	81.32	86.86	91.20

#### 4.3.2 Support vector machine model

**FIGURE 3:**
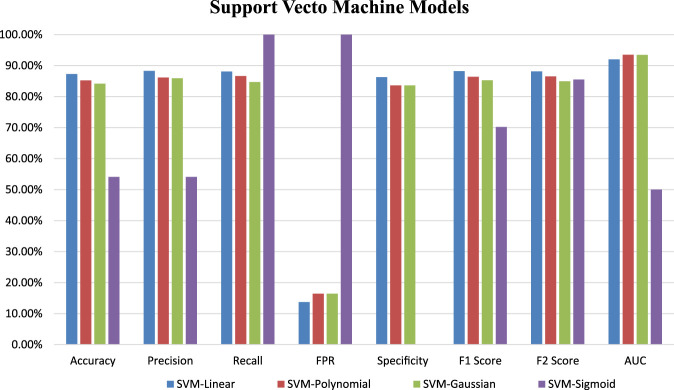
Graphical representation of developed SVM models performance based on evaluation parameters.

**FIGURE 4:**
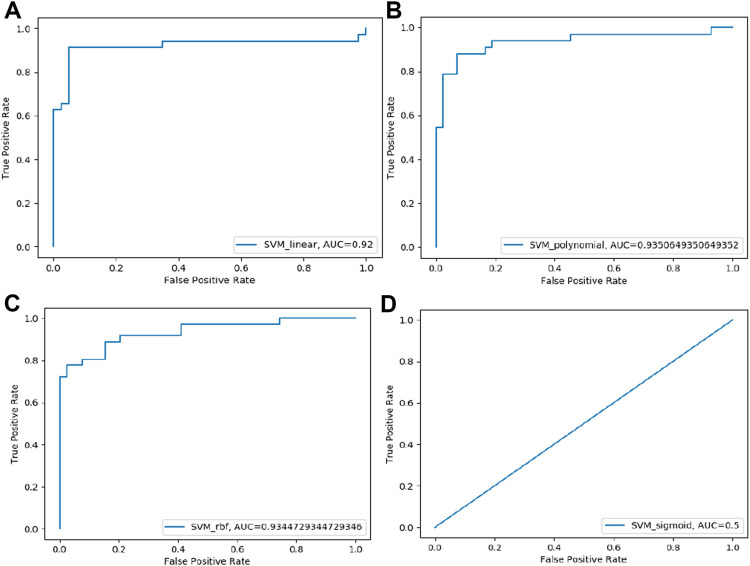
ROC curve of four SVM models performance based on AUC score: **(A)** SVM-Linear model ROC curve **(B)** SVM-Polynomial model ROC curve **(C)** SVM-rbf model ROC curve **(D)** SVM-Sigmoid model ROC curve.

Four SVM models were trained on training datasets and the performance of each of the trained models was evaluated on test data set by using several evaluation parameters. For validation purposes in this paper, we used k-Fold i.e., *k* = 5 cross-validation techniques. In this paper, we compare all four developed SVM models with each other based on their evaluation parameters (Table 4). The SVM_Linear model gives better accuracy (87.26%) and precision (88.31%) in comparison to other models. So, among all the developed SVM models the SVM-Linear model performs very well compared to the other three models (SVM-Polynomial, SVM-Gaussian, SVM-Sigmoid). The values of all six evaluation parameters have been plotted against the undertaken SVM models (Figures 3, 4) which show similar comparisons in a graphical format for easy understanding. This study fits under the TOPSIS (Technique for Order Preference by Similarity to Ideal Solution) framework (Hwang and Yoon 1981). According to TOPSIS analysis result SVM.Linear model got 1st rank (Supplementary Material).

**TABLE 4:** Comparison of developed SVM models performance based on evaluation parameters (figures in percentage).

Models	Accuracy	Precision	Recall	FPR	Specificity	F1 score	F2 score	AUC
SVM-Linear	87.26	88.31	88.10	13.74	86.26	88.21	88.14	92.00
SVM-Polynomial	85.22	86.15	86.61	16.41	83.59	86.38	86.52	93.51
SVM-Gaussian	84.18	85.88	84.68	16.41	83.59	85.28	84.92	93.45
SVM-Sigmoid	54.09	54.09	100	100	0.00	70.21	85.49	50.00

#### 4.3.3 Random forest model

**FIGURE 5:**
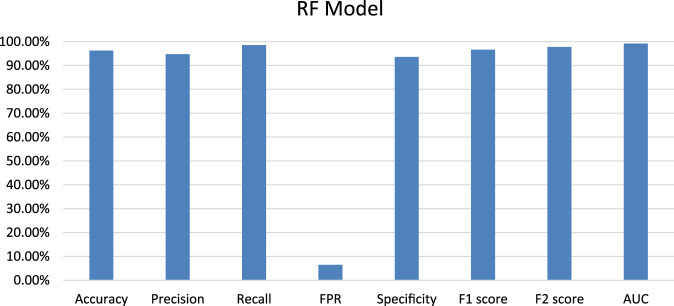
Graphical representation of developed Random Forest model performance based on evaluation parameters.

**FIGURE 6:**
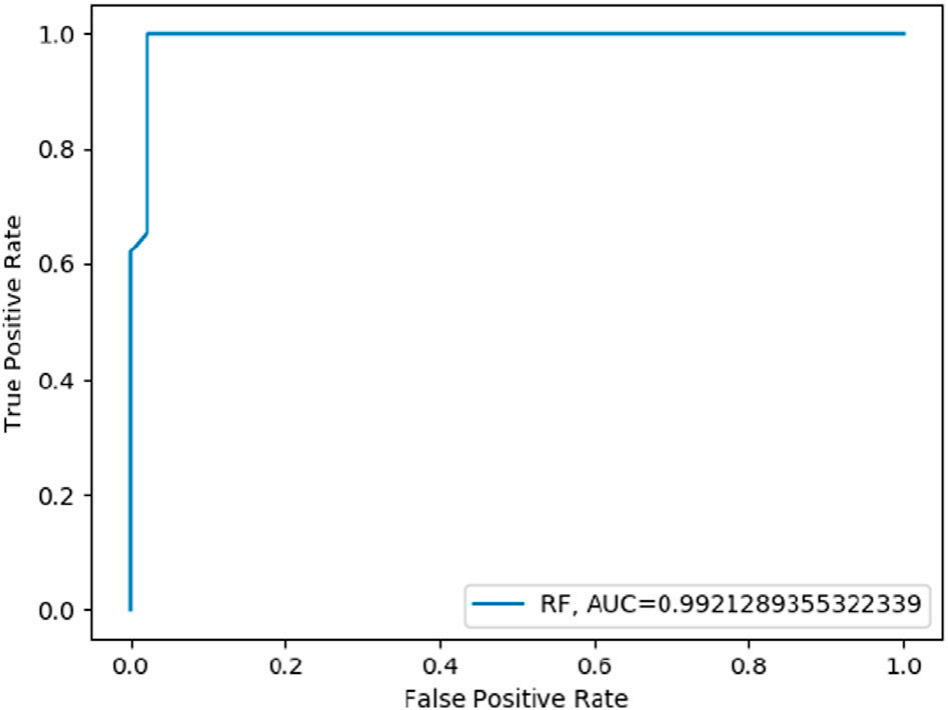
ROC curve of RF model performance based on AUC score.

Random Forest (RF) model was trained on training datasets and the performance of the trained model was evaluated on test data set by using several evaluation parameters. For validation purposes in this paper, we used k-Fold i.e., *k* = 5 cross-validation techniques. The value of RF model accuracy is 96.27% and its AUC value is 99.21% (Table 5). The graphical representation of the model performance is also shown in Figure 5. Here, the RF model gives a very low score of false positive rate (FPR), which is good for any model. From the ROC curve of the RF model (Figure 6) it can be concluded that the developed model is performing very well in the plant data set.

**TABLE 5:** Different performance parameters of the random forest model (figures in percentage).

Parameters	RF model
Accuracy	96.27
Precision	94.75
Recall	98.56
FPR	6.44
Specificity	93.56
F1 score	96.62
F2 score	97.77
AUC	99.21

### 4.4 Comparison among developed machine learning-based models

**FIGURE 7:**
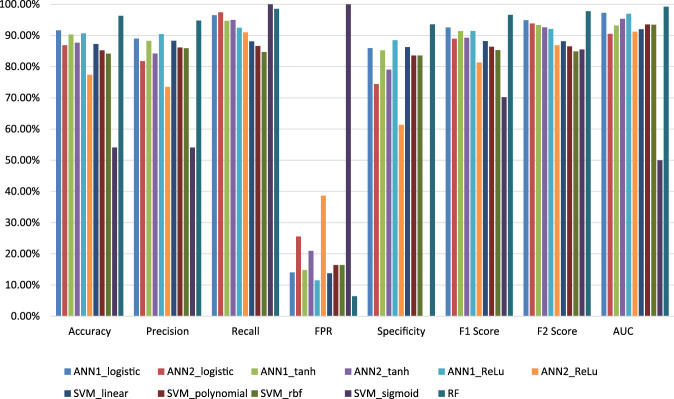
Graphical representation of the performance of all models for off-target prediction developed using three groups of machine learning techniques.

In this study, we developed three machine learning-based models for the estimation of off-target sites. The performance of these techniques is being evaluated by different statistical measures viz. sensitivity/recall, specificity, accuracy, precision, FPR, F1 score, F2 score and AUC (Table 6). Random Forest (RF) model achieves the best accuracy which is 96.27% compare to other models. RF model achieves the highest specificity value as compared to the other ten models which are 93.56%. According to the AUC score RF model cover, the maximum area under the ROC curve is 99.21% compare to the other seven models (Figure 7). From the above comparisons, Random Forest (RF) model perform comparatively better than the other ten models on plant datasets and got 1st rank according to TOPSIS analysis (Table 7).

**TABLE 6:** Comparison of all eleven developed models performance based on evaluation parameters (figures in percentage).

	ANN1_logistic	ANN2_logistic	ANN1_tanh	ANN2_tanh	ANN1_ReLu	ANN2_ReLu	SVM_linear	SVM_polynomial	SVM_rbf	SVM_sigmoid	Random forest
Accuracy	91.65	86.87	90.33	87.66	90.65	77.39	87.26	85.22	84.18	54.09	96.27
Precision	89.00	81.78	88.29	84.23	90.44	73.50	88.31	86.15	85.88	54.09	94.75
Recall	96.49	97.44	94.68	94.98	92.49	91.00	88.10	86.61	84.68	100.00	98.56
FPR	14.05	25.57	14.80	20.95	11.52	38.65	13.74	16.41	16.41	100.00	6.44
Specificity	85.95	74.43	85.20	79.05	88.48	61.35	86.26	83.59	83.59	0.00	93.56
F1 Score	92.59	88.93	91.37	89.28	91.45	81.32	88.21	86.38	85.28	70.21	96.62
F2 Score	94.89	93.85	93.33	92.61	92.07	86.86	88.14	86.52	84.92	85.49	97.77
AUC	97.26	90.54	93.18	95.34	96.94	91.20	92.00	93.51	93.45	50.00	99.21

**TABLE 7:** TOPSIS analysis result for all eleven machine learning-developed models on the plant dataset.

Models	Score	Rank
RF	0.961423	1
ANN1_logistic	0.789202	2
ANN1_ReLu	0.69774	3
ANN1_tanh	0.602147	4
ANN2_tanh	0.516367	5
SVM_linear	0.482942	6
ANN2_logistic	0.447746	7
SVM_polynomial	0.386691	8
SVM_rbf	0.327762	9
SVM_sigmoid	0.276047	10
ANN2_ReLu	0.166041	11

## 5 Discussion

In CRISPR-Cas9 gene editing techniques, sgRNA seeks to target a specific DNA segment but occasionally it may attach to untargeted DNA locations, which regrettably results in off-target mutations. Altering gene functionalities brought on by these off-target mutations might generate significant genomic instability and constitute a serious concern. This off-target induced resultant genomic instability causes a major limitation in the use of the CRISPR-Cas9 gene editing technique. Therefore, it is essential to accurately predict the off-target site related to sgRNA.

In this study, we used a machine learning approach for predicting off-targets in the CRISPR-Cas9 gene editing technique. Here, a total of eleven machine learning-based models for CRISPR-Cas9 cleavage site prediction (6-ANN, 4-SVM, and 1-RF models) were constructed. Models were trained by using three main types of features pair-wise alignment features from the sgRNA-DNA sequence, features related to nucleotide composition and PAM site characteristics. Training of the various machine learning algorithms viz. ANN, SVM and RF were carried out with different combinations of layer counts, kernel types and tree counts respectively. The performance of the training models was evaluated based on selected statistics within and between groups of developed models.

In the case of Artificial Neural Networks (ANN), six models were developed using different activation functions, different hidden layers and different neuron numbers. Following activation functions Logistic, Tanh and ReLU has been applied with varying number of layers and neurons resulting in six ANN-based models named as ANN1-Logistic, ANN2-Logistic, ANN1-Tanh, ANN2-Tanh, ANN1-ReLU and ANN2-ReLU. These models were trained on plant data sets and performance was evaluated under 5-fold cross-validation. Although, they have achieved more or less similar performance but according to TOPSIS analysis ANN1-Logistic model predicts off-target sites accurately as compared to the other five models *i.e.* ANN2-Logistic, ANN1-Tanh, ANN2-Tanh, ANN1-ReLU and ANN2-ReLU.

In the instance of a Support Vector Machine (SVM), a total of four models were developed using different kernel functions. The following kernel functions: Linear, Polynomial, Gaussian and Sigmoid have been used during the model training. Which results in four SVM-based models named as SVM-Linear, SVM-Polynomial, SVM-Gaussian and SVM-Sigmoid. These models were trained using data sets related to plants. The relative performance among the SVM-based models was evaluated using 5-fold cross-validation and TOPSIS analysis. Based on these evaluations, the SVM-Linear model predicts off-target sites more accurately than the other three SVM models i.e., SVM-Polynomial, SVM-Gaussian, and SVM-Sigmoid.

Random Forest (RF) experiments were carried out with different numbers of tree sizes and a model with optimal tree size was selected for further comparison with other developed models.

We evaluated the relative accuracy of the three groups of developed machine learning-based models for off-target prediction, using the 5-fold cross-validation method and TOPSIS analysis. The accuracy of the RF model was 96.27%, and its area under the ROC curve (AUC) was 99.21%, which is higher than that of the ANN and SVM models. Further, based on the TOPSIS analysis, the Random Forest model was scored highest among the group. This indicates the better performance of the Random Forest model over SVM and ANN-based models for the prediction of cleavage sites in the CRISPR-Cas9 gene editing technique for plant systems.

In the future, the latest machine-learning techniques such as deep learning etc. may be investigated further to enhance of the modeling process. It is generally accepted that such cutting-edge, computationally intelligent strategies will make future predictions of CRISPR-Cas9 off-target sites even more accurate.

## 6 Conclusion

Gene editing, commonly known as molecular scissors, is the process of insertion, deletion or replacement of DNA on a particular position in the genome of any organism. We demonstrated that the off-targets of CRISPR-Cas9 gene editing can be reliably predicted by machine learning approaches. In comparison to the other two conventional machine learning methods, ANN and SVM; our final Random Forest (RF) model better them all in terms of performance on the plant dataset. We think that these intelligent methods can make a significant contribution to CRISPR-Cas9 off-target predictions or other problems of a similar nature.

## Data Availability

The data analyzed in this study is subject to the following licenses/restrictions: Dataset will be made available on request. Requests to access these datasets should be directed to jutandas2468@gmail.com.

## References

[B1] Abadi S. Yan W. X. Amar D. Mayrose I. (2017). A machine learning approach for predicting CRISPR-Cas9 cleavage efficiencies and patterns underlying its mechanism of action. PLOS Comput. Biol. 13 (10), e1005807. 10.1371/journal.pcbi.1005807 29036168 PMC5658169

[B2] Andersson M. Turesson H. Nicolia A. Fält A. S. Samuelsson M. Hofvander P. (2017). Efficient targeted multiallelic mutagenesis in tetraploid potato (Solanum tuberosum) by transient CRISPR-Cas9 expression in protoplasts. Plant Cell. Rep. 36 (1), 117–128. 10.1007/s00299-016-2062-3 27699473 PMC5206254

[B3] Barrangou R. Fremaux C. Deveau H. Richards M. Boyaval P. Moineau S. (2007). CRISPR provides acquired resistance against viruses in prokaryotes. Science 315 (5819), 1709–1712. 10.1126/science.1138140 17379808

[B4] Bortesi L. Fischer R. (2015). The CRISPR/Cas9 system for plant genome editing and beyond. Biotechnol. Adv. 33 (1), 41–52. 10.1016/j.biotechadv.2014.12.006 25536441

[B5] Breslauer K. J. Frank R. Blocker H. Marky L. A. (1986). Predicting DNA duplex stability from the base sequence. Proc. Natl. Acad. Sci. 83 (11), 3746–3750. 10.1073/pnas.83.11.3746 3459152 PMC323600

[B6] Brooks C. Nekrasov V. Lipppman Z. B. van Eck J. (2014). Efficient gene editing in tomato in the first generation using the clustered regularly interspaced short palindromic repeats/CRISPR-associated9 system. Plant Physiol. 166 (3), 1292–1297. 10.1104/pp.114.247577 25225186 PMC4226363

[B7] Butler N. M. Atkins P. A. Voytas D. F. Douches D. S. (2015). Generation and inheritance of targeted mutations in potato (Solanum tuberosum L.) using the CRISPR/cas system. PLOS ONE 10 (12), e0144591. 10.1371/journal.pone.0144591 26657719 PMC4684367

[B8] Cai Y. Chen L. Liu X. Sun S. Wu C. Jiang B. (2015). CRISPR/Cas9-Mediated genome editing in soybean hairy roots. PLOS ONE 10 (8), e0136064. 10.1371/journal.pone.0136064 26284791 PMC4540462

[B9] Čermák T. Baltes N. J. Čegan R. Zhang Y. Voytas D. F. (2015). High-frequency, precise modification of the tomato genome. Genome Biol. 16 (1), 232–315. 10.1186/s13059-015-0796-9 26541286 PMC4635538

[B10] Chandrasekaran J. Brumin M. Wolf D. Leibman D. Klap C. Pearlsman M. (2016). Development of broad virus resistance in non-transgenic cucumber using CRISPR/Cas9 technology. Mol. Plant Pathol. 17 (7), 1140–1153. 10.1111/mpp.12375 26808139 PMC6638350

[B11] Chatterjee A. Saha J. Mukherjee J. (2022). Clustering with multi-layered perceptron. Pattern Recognit. Lett. 155, 92–99. 10.1016/J.PATREC.2022.02.009

[B12] Chen X. Lu X. Shu N. Wang S. Wang J. Wang D. (2017). Targeted mutagenesis in cotton (Gossypium hirsutum L.) using the CRISPR/Cas9 system. Sci. Rep. 7 (1), 44304–44307. 10.1038/srep44304 28287154 PMC5347080

[B13] Cho S. W. Kim S. Kim Y. Kweon J. Kim H. S. Bae S. (2014). Analysis of off-target effects of CRISPR/Cas-derived RNA-guided endonucleases and nickases. Genome Res. 24 (1), 132–141. 10.1101/gr.162339.113 24253446 PMC3875854

[B14] Doench J. G. Fusi N. Sullender M. Hegde M. Vaimberg E. W. Donovan K. F. (2016). Optimized sgRNA design to maximize activity and minimize off-target effects of CRISPR-Cas9. Nat. Biotechnol. 34 (2), 184–191. 10.1038/nbt.3437 26780180 PMC4744125

[B15] Feng C. Su H. Bai H. Wang R. Liu Y. Guo X. (2018). High-efficiency genome editing using a dmc1 promoter-controlled CRISPR/Cas9 system in maize. Plant Biotechnol. J. 16 (11), 1848–1857. 29569825 10.1111/pbi.12920 PMC6181213

[B16] Feng C. Yuan J. Wang R. Liu Y. Birchler J. A. Han F. (2016). Efficient targeted genome modification in maize using CRISPR/Cas9 system. J. Genet. Genomics 43 (1), 37–43. 10.1016/J.JGG.2015.10.002 26842992

[B17] Gao J. Wang G. Ma S. Xie X. Wu X. Zhang X. (2014). CRISPR/Cas9-mediated targeted mutagenesis in Nicotiana tabacum. Plant Mol. Biol. 87 (1), 99–110. 10.1007/s11103-014-0263-0 25344637

[B18] Gao W. Long L. Tian X. Xu F. Liu J. Singh P. K. (2017). Genome editing in cotton with the CRISPR/Cas9 system. Front. Plant Sci. 8, 1364. 10.3389/fpls.2017.01364 28824692 PMC5541054

[B19] Georges F. Ray H. (2017). Genome editing of crops: A renewed opportunity for food security. Taylor Francis 8 (1), 1–12. 10.1080/21645698.2016.1270489 PMC5592977 28075688

[B20] Grinblat G. L. Uzal L. C. Larese M. G. Granitto P. M. (2016). Deep learning for plant identification using vein morphological patterns. Comput. Electron. Agric. 127, 418–424. 10.1016/J.COMPAG.2016.07.003

[B21] Haeussler M. Schonig K. Eckert H. Eschstruth A. Mianne J. Renaud J. B. (2016). Evaluation of off-target and on-target scoring algorithms and integration into the guide RNA selection tool CRISPOR. Genome Biol. 17 (1), 148–212. 10.1186/s13059-016-1012-2 27380939 PMC4934014

[B22] Haque E. Taniguchi H. Hassan M. M. Bhowmik P. Karim M. R. Śmiech M. (2018). Application of CRISPR/Cas9 genome editing technology for the improvement of crops cultivated in tropical climates: Recent progress, prospects, and challenges. Front. Plant Sci. 9, 617. 10.3389/fpls.2018.00617 29868073 PMC5952327

[B23] Hesami M. Alizadeh M. Jones A. M. P. Torkamaneh D. (2022). Machine learning: Its challenges and opportunities in plant system biology. Appl. Microbiol. Biotechnol. 106 (9), 3507–3530. 10.1007/s00253-022-11963-6 35575915

[B24] Hesami M. Jones A. M. P. (2020). Application of artificial intelligence models and optimization algorithms in plant cell and tissue culture. Appl. Microbiol. Biotechnol. 104 (22), 9449–9485. 10.1007/s00253-020-10888-2 32984921

[B25] Hesami M. Yoosefzadeh Najafabadi M. Adamek K. Torkamaneh D. Jones A. M. P. (2021). Synergizing off-target predictions for in silico insights of CENH3 knockout in cannabis through CRISPR/cas. Molecules 26 (7), 2053. 10.3390/molecules26072053 33916717 PMC8038328

[B26] Hsu P. D. Scott D. A. Weinstein J. A. Ran F. A. Konermann S. Agarwala V. (2013). DNA targeting specificity of RNA-guided Cas9 nucleases. Nat. Biotechnol. 31 (9), 827–832. 10.1038/nbt.2647 23873081 PMC3969858

[B27] Hwang C.-L. Yoon K. (1981). “Methods for multiple attribute decision making,” in Multiple attribute decision making. Lecture notes in economics and mathematical systems (Berlin, Heidelberg: Springer), Vol. 186, 58–191. 10.1007/978-3-642-48318-9_3

[B28] Jafari M. Shahsavar A. (2020). The application of artificial neural networks in modeling and predicting the effects of melatonin on morphological responses of citrus to drought stress. PLOS ONE 15 (10), e0240427. 10.1371/journal.pone.0240427 33052940 PMC7556499

[B29] Jia H. Nian W. (2014). Targeted genome editing of sweet orange using cas9/sgRNA. PLOS ONE 9 (4), e93806. 10.1371/journal.pone.0093806 24710347 PMC3977896

[B30] Kapusi E. Corcuera-Gómez M. Melnik S. Stoger E. (2017). Heritable genomic fragment deletions and small indels in the putative ENGase gene induced by CRISPR/Cas9 in barley. Front. Plant Sci. 8, 540. 10.3389/fpls.2017.00540 28487703 PMC5404177

[B31] Kim D. Alptekin B. Budak H. (2018). CRISPR/Cas9 genome editing in wheat. Funct. Integr. Genomics 18 (1), 31–41. 10.1007/s10142-017-0572-x 28918562

[B32] Li C. Unver T. Zhang B. (2017a2017). A high-efficiency CRISPR/Cas9 system for targeted mutagenesis in Cotton (Gossypium hirsutum L.). Sci. Rep. 7 (1), 43902–43910. 10.1038/srep43902 PMC5335549 28256588

[B33] Li J. Meng X. Zong Y. Chen K. Zhang H. Liu J. (2016). Gene replacements and insertions in rice by intron targeting using CRISPR–Cas9. Nat. Plants 2 (10), 16139–16145. 10.1038/nplants.2016.139 27618611

[B34] Li J. Sun Y. Du J. Zhao Y. Xia L. (2017b). Generation of targeted point mutations in rice by a modified CRISPR/Cas9 system. Mol. Plant 10 (3), 526–529. 10.1016/j.molp.2016.12.001 27940306

[B35] Lin J. Wong K. C. (2018). Off-target predictions in CRISPR-Cas9 gene editing using deep learning. Bioinformatics 34 (17), i656–i663. 10.1093/bioinformatics/bty554 30423072 PMC6129261

[B36] Ma H. Marti-Gutierrez N. Park S. W. Wu J. Lee Y. Suzuki K. (2017). Correction of a pathogenic gene mutation in human embryos. Nature 548 (7668), 413–419. 10.1038/nature23305 28783728

[B37] Malnoy M. Viola R. Jung M. H. Koo O. J. Kim S. Kim J. S. (2016). DNA-free genetically edited grapevine and apple protoplast using CRISPR/Cas9 ribonucleoproteins. Front. Plant Sci. 7 (12), 1904. 10.3389/fpls.2016.01904 28066464 PMC5170842

[B38] Martín-Pizarro C. Triviño J. C. Posé D. (2019). Functional analysis of the TM6 MADS-box gene in the octoploid strawberry by CRISPR/Cas9-directed mutagenesis. J. Exp. Bot. 70 (3), 885–895. 10.1093/jxb/ery400 30428077 PMC6363087

[B39] Mishra B. Kumar N. Mukhtar M. S. (2019). Systems biology and machine learning in plant–pathogen interactions. Mol. Plant-Microbe Interact. 32 (1), 45–55. 10.1094/MPMI-08-18-0221-FI 30418085

[B40] Mitchell T. Buchanan B. Dejong G. Dietterich T. Rosenbloom P. Waibel A. (2003). Machine learning. Annu. Rev. Comput. Sci. 4 (1), 417–433. 10.1146/annurev.cs.04.060190.002221

[B41] Musunuru K. (2017). Genome editing: The recent history and perspective in cardiovascular diseases. J. Am. Coll. Cardiol. 70 (22), 2808–2821. 10.1016/J.JACC.2017.10.002 29191331 PMC5742864

[B42] Nakajima I. Ban Y. Azuma A. Onoue N. Moriguchi T. Yamamoto T. (2017). CRISPR/Cas9-mediated targeted mutagenesis in grape. PLOS ONE 12 (5), e0177966. 10.1371/journal.pone.0177966 28542349 PMC5436839

[B43] Niu M. Lin Y. Zou Q. (2021). sgRNACNN: identifying sgRNA on-target activity in four crops using ensembles of convolutional neural networks. Plant Mol. Biol. 105 (4), 483–495. 10.1007/s11103-020-01102-y 33385273

[B44] Pan C. Ye L. Qin L. Liu X. He Y. Wang J. (2016). CRISPR/Cas9-mediated efficient and heritable targeted mutagenesis in tomato plants in the first and later generations. Sci. Rep. 6 (1), 24765–24769. 10.1038/srep24765 27097775 PMC4838866

[B45] Pedregosa F. (2011). Scikit-learn: Machine learning in Python gaël varoquaux bertrand thirion vincent dubourg alexandre passos PEDREGOSA, VAROQUAUX, GRAMFORT ET AL. Matthieu perrot. J. Mach. Learn. Res. 12, 2825–2830. Available at: http://scikit-learn.sourceforge.net (Accessed November 6, 2022).

[B46] Perez-Pinera P. Ousterout D. G. Gersbach C. A. (2012). Advances in targeted genome editing. Curr. Opin. Chem. Biol. 16 (3–4), 268–277. 10.1016/j.cbpa.2012.06.007 22819644 PMC3424393

[B47] Refaeilzadeh P. Tang L. Liu H. (2009). Cross-validation. Encycl. Database Syst. 5, 532–538. 10.1007/978-0-387-39940-9_565

[B48] Sander J. D. Joung J. K. (2014). CRISPR-Cas systems for editing, regulating and targeting genomes. Nat. Biotechnol. 32 (4), 347–355. 10.1038/nbt.2842 24584096 PMC4022601

[B49] Sanjana N. E. Shalem O. Zhang F. (2014). Improved vectors and genome-wide libraries for CRISPR screening. Nat. Methods 11 (8), 783–784. 10.1038/nmeth.3047 25075903 PMC4486245

[B50] Shan Q. Wang Y. Li J. Gao C. (2014). Genome editing in rice and wheat using the CRISPR/Cas system. Nat. Protoc. 9 (10), 2395–2410. 10.1038/nprot.2014.157 25232936

[B51] Singh A. Ganapathysubramanian B. Singh A. K. Sarkar S. (2016). Machine learning for high-throughput stress phenotyping in plants. Trends Plant Sci. 21 (2), 110–124. 10.1016/J.TPLANTS.2015.10.015 26651918

[B52] Sovová T. Kerins G. Demnerová K. Ovesná J. (2016). Genome editing with engineered nucleases in economically important animals and plants: State of the art in the research pipeline. Curr. Issues Mol. Biol. 21, 41–62. 27253613

[B53] Stemmer M. Thumberger T. del Sol Keyer M. Wittbrodt J. Mateo J. L. (2015). CCTop: An intuitive, flexible and reliable CRISPR/Cas9 target prediction tool. PLOS ONE 10 (4), e0124633. 10.1371/journal.pone.0124633 25909470 PMC4409221

[B54] Sun X. Hu Z. Chen R. Jiang Q. Song G. Zhang H. (2015). Targeted mutagenesis in soybean using the CRISPR-Cas9 system. Sci. Rep. 5 (1), 10342–10410. 10.1038/srep10342 26022141 PMC4448504

[B55] Svitashev S. Schwartz C. Lenderts B. Young J. K. Mark Cigan A. (2016). Genome editing in maize directed by CRISPR–Cas9 ribonucleoprotein complexes. Nat. Commun. 7 (1), 13274–13277. 10.1038/ncomms13274 27848933 PMC5116081

[B56] Terns M. P. Terns R. M. (2011). CRISPR-based adaptive immune systems. Curr. Opin. Microbiol. 14 (3), 321–327. 10.1016/J.MIB.2011.03.005 21531607 PMC3119747

[B57] Tian S. Jiang L. Gao Q. Zhang J. Zong M. Zhang H. (2016). Efficient CRISPR/Cas9-based gene knockout in watermelon. Plant Cell. Rep. 36 (3), 399–406. 10.1007/s00299-016-2089-5 27995308

[B58] Urnov F. D. Rebar E. J. Holmes M. C. Zhang H. S. Gregory P. D. (2010). Genome editing with engineered zinc finger nucleases. Nat. Rev. Genet. 11 (9), 636–646. 10.1038/nrg2842 20717154

[B59] van Dijk A. D. J. Kootstra G. Kruijer W. de Ridder D. (2021). Machine learning in plant science and plant breeding. iScience 24 (1), 101890. 10.1016/J.ISCI.2020.101890 33364579 PMC7750553

[B60] Wang M. Mao Y. Lu Y. Tao X. Zhu J. (2017). Multiplex gene editing in rice using the CRISPR-cpf1 system. Mol. Plant 10, 1011–1013. 10.1016/j.molp.2017.03.001 28315752

[B61] Wang P. Zhang J. Sun L. Ma Y. Xu J. Liang S. (2018). High efficient multisites genome editing in allotetraploid cotton (Gossypium hirsutum) using CRISPR/Cas9 system. Plant Biotechnol. J. 16 (1), 137–150. 10.1111/pbi.12755 28499063 PMC5785356

[B62] Wang S. Zhang S. Wang W. Xiong X. Meng F. Cui X. (2015). Efficient targeted mutagenesis in potato by the CRISPR/Cas9 system. Plant Cell. Rep. 34 (9), 1473–1476. 10.1007/s00299-015-1816-7 26082432

[B63] Westra E. R. Buckling A. Fineran P. C. (2014). CRISPR-cas systems: Beyond adaptive immunity. Nat. Rev. Microbiol. 12 (5), 317–326. 10.1038/nrmicro3241 24704746

[B64] Wood A. J. Lo T. W. Zeitler B. Pickle C. S. Ralston E. J. Lee A. H. (2011). Targeted genome editing across species using ZFNs and TALENs. Science 333 (6040), 307. 21700836 10.1126/science.1207773 PMC3489282

[B65] Xu R. F. Li H. Qin R. Y. Li J. Qiu C. H. Yang Y. C. (2015). Generation of inheritable and “transgene clean” targeted genome-modified rice in later generations using the CRISPR/Cas9 system. Sci. Rep. 5 (1), 11491–11501. 10.1038/srep11491 26089199 PMC5155577

[B66] Xu R. Li H. Qin R. Wang L. Li L. Wei P. (2014). Gene targeting using the Agrobacterium tumefaciens-mediated CRISPR-Cas system in rice. Rice 7 (1), 5–4. 10.1186/s12284-014-0005-6 24920971 PMC4052633

[B67] Xu X. Duan D. Chen S. J. (2017). CRISPR-Cas9 cleavage efficiency correlates strongly with target-sgRNA folding stability: From physical mechanism to off-target assessment. Sci. Rep. 7 (1), 143–152. 10.1038/s41598-017-00180-1 28273945 PMC5427927

[B68] Zhang H. Zhang J. Wei P. Zhang B. Gou F. Feng Z. (2014). The CRISPR/Cas9 system produces specific and homozygous targeted gene editing in rice in one generation. Plant Biotechnol. J. 12 (6), 797–807. 10.1111/pbi.12200 24854982

[B69] Zhang Y. Liang Z. Zong Y. Wang Y. Liu J. Chen K. (2016). Efficient and transgene-free genome editing in wheat through transient expression of CRISPR/Cas9 DNA or RNA. Nat. Commun. 7 (1), 12617–12618. 10.1038/ncomms12617 27558837 PMC5007326

[B70] Zhou H. Liu B. Weeks D. P. Spalding M. H. Yang B. (2014). Large chromosomal deletions and heritable small genetic changes induced by CRISPR/Cas9 in rice. Nucleic Acids Res. 42 (17), 10903–10914. 10.1093/nar/gku806 25200087 PMC4176183

[B71] Zhou T. Yang L. Lu Y. Dror I. Dantas Machado A. C. Ghane T. (2013). DNAshape: A method for the high-throughput prediction of DNA structural features on a genomic scale. Nucleic Acids Res. 41 (W1), W56–W62. 10.1093/nar/gkt437 23703209 PMC3692085

