# Supplementary material for: Machine learning in the estimation of CRISPR-Cas9 cleavage sites for plant system
Source: Front Genet. 2023 Jan 9;13:1085332. doi: 10.3389/fgene.2022.1085332 (PMC9868961; doi:10.3389/fgene.2022.1085332)
Supplement: Supplementary file 1 [file DataSheet1.docx]

**Supplementary Information**

**Table-1** SVM models TOPSIS analysis results

| **models** | **alt.row** | **score** | **rank** |
| --- | --- | --- | --- |
| SVM.Linear | 1 | 0.76155 | 1 |
| SVM.Polynomial | 2 | 0.58102 | 2 |
| SVM.Gaussian | 3 | 0.373309 | 3 |
| SVM.Sigmoid | 4 | 0.288013 | 4 |

**Table-2** ANN models TOPSIS analysis results

| **models** | **alt.row** | **score** | **rank** |
| --- | --- | --- | --- |
| ANN1.Logistic | 1 | 0.85134 | 1 |
| ANN1.ReLU | 5 | 0.671288 | 2 |
| ANN1.Tanh | 3 | 0.554693 | 3 |
| ANN2.Tanh | 4 | 0.445307 | 4 |
| ANN2.Logistic | 2 | 0.403155 | 5 |
| ANN2.ReLU | 6 | 0 | 6 |
